# Reg proteins promote acinar-to-ductal metaplasia and act as novel diagnostic and prognostic markers in pancreatic ductal adenocarcinoma

**DOI:** 10.18632/oncotarget.12834

**Published:** 2016-10-24

**Authors:** Qing Li, Hao Wang, George Zogopoulos, Qin Shao, Kun Dong, Fudong Lv, Karam Nwilati, Xian-yong Gui, Adeline Cuggia, Jun-Li Liu, Zu-hua Gao

**Affiliations:** ^1^ Fraser Laboratories for Diabetes Research, Department of Medicine, McGill University Health Centre, Montreal, QC, Canada; ^2^ Department of Oncology, Qingdao Municipal Hospital, School of Medicine, Qingdao University, Qingdao, China; ^3^ Department of Surgery, McGill University Health Centre, Montreal, QC, Canada; ^4^ Quebec Pancreas Cancer Study, McGill University Health Centre, Montreal, QC, Canada; ^5^ Department of Pathology, McGill University Health Centre, Montreal, QC, Canada; ^6^ Department of Pathology, You An Hospital, Capital Medical University, Beijing, China; ^7^ Department of Pathology, University of Calgary, Calgary, AB, Canada

**Keywords:** Reg family proteins, pancreatic ductal adenocarcinoma, acinar-to-ductal metaplasia, pancreatic intraepithelial neoplasia, cholangiocarcinoma

## Abstract

Pancreatic ductal adenocarcinoma (PDAC) is a highly aggressive malignant tumor. Acinar-to-ductal metaplasia (ADM) and pancreatic intraepithelial neoplasia (PanIN) are both precursor lesions that lead to the development of PDAC. Reg family proteins (Reg1A, 1B, 3A/G, 4) are a group of calcium-dependent lectins that promote islet growth in response to inflammation and/or injuries. The aim of this study was to establish a role for Reg proteins in the development of PDAC and their clinical value as biomarkers. We found that Reg1A and Reg3A/G were highly expressed in the ADM tissues by immunohistochemistry. In the 3-dimensional culture of mouse acinar cells, Reg3A promoted ADM formation with concurrent activation of mitogen-acitvated protein kinase. Upregulation of Reg1A and Reg1B levels was observed as benign ductal epithelium progresses from PanIN to invasive PDAC. Patients with PDAC showed significantly higher serum levels of Reg1A and Reg1B than matching healthy subjects. These results were further validated by the quantification of Reg 1A and 1B mRNA levels in the microdissected tissues (22- and 6-fold increases vs. non-tumor tissues). Interestingly, patients with higher levels of Reg1A and 1B exhibited improved survival rate than those with lower levels. Furthermore, tissue expressions of Reg1A, Reg1B, and Reg4 could differentiate metastatic PDAC in the liver from intrahepatic cholangiocarcinoma with 92% sensitivity and 95% specificity. Overall, our results demonstrate the upregulation of Reg proteins during PDAC development. If validated in larger scale, Reg1A and Reg1B could become clinical markers for detecting early stages of PDAC, monitoring therapeutic response, and/or predicting patient's prognosis.

## INTRODUCTION

Pancreatic ductal adenocarcinoma (PDAC) is the fourth leading cause of cancer-related death [1]. Traditionally, it is believed that PDAC starts from a distinct precursor lesion named pancreatic intraepithelial neoplasia (PanIN) and progresses to invasive carcinoma through a series of genetic events. The activation of the K-ras oncogene and inactivation of tumor suppressor genes including CDKN2A and TP53, and transcriptional factor SMAD4/DPC4 have all been implicated [2]. Recent studies suggest that PDAC can also derive from acinar-to-ductal metaplasia (ADM), with additional mutations in K-ras and TP53 [3, 4]. ADM is also a protective mechanism of acinar cells in response to inflammatory stimuli, such as chronic pancreatitis or interleukin-17 [5, 6].

Due to the deep anatomical location of the human pancreas, tumor-specific symptoms of PDAC, such as abdominal mass, jaundice, and weight loss, typically emerge only after the tumor has reached advanced stages. It is either unresectable or has already metastasized to the liver or other organs [7]. In order to implement an effective therapy and improve patients' prognosis, sensitive and specific biomarkers to aid in early diagnosis are urgently needed. Moreover, when PDAC metastasizes to the liver, it needs to be differentiated from primary intrahepatic cholangiocarcinoma (ICA). The therapeutic approaches and prognoses for ICA and metastatic PDAC in the liver are completely different. Surgery is the primary therapeutic option with a 5-year survival rate up to 40% for patients with resectable ICA [8]. Metastatic PDAC, however, is usually unresectable and the treatment option is limited to palliative chemoradiotherapy. This clinical demand poses a huge challenge to surgical pathologists because the histomorphological and immunohistochemical profiles of ICA and PDAC are essentially identical. Therefore, clinically applicable biomarkers that can clearly differentiate these two malignant tumors are needed to guide appropriate therapies and provide more accurate staging information for predicting patient prognosis.

The family of Regenerating (Reg) proteins is a group of C-type lectin-like proteins discovered in patients with pancreatitis and during pancreatic islet regeneration [9]. Five Reg family members including Reg1A, Reg1B, Reg3A, Reg3G and Reg4 have been identified in humans. The overexpression of the Reg1A gene in pancreatic cancer cells has been shown to result in accelerated cell proliferation and tumor growth, both *in vitro* and *in vivo* [10]. Reg3 subfamily, including Reg3A and Reg3G, are known as pancreatitis-associated proteins due to their activation in response to inflammatory stimuli [11]. Recently, Reg3A has been reported to accelerate pancreatic cancer cell growth in response to interlukin-6 via the JAK2/STAT3 signaling pathway [12, 13]. Reg4, the most recently discovered member of the family, was reported to be elevated in PDAC and proposed to be a diagnostic marker [14, 15]. Moreover, a proteomic analysis of pancreatic juice demonstrated increased levels of Reg1A, Reg1B and Reg3A proteins in PDAC, in comparison to normal subjects and patients with pancreatitis [16]. However, the involvement of Reg proteins in the onset and progression of PDAC has yet to be elucidated. In the present study, we first demonstrated the presence of Reg proteins in precursors to PDAC, including ADM and PanIN. We then evaluated the diagnostic and prognostic value of Reg proteins in PDAC by measuring the serum levels and tissue expression of Reg proteins in association with the malignant progression of PDAC and patients' prognoses. Lastly, we assessed the role of Reg proteins in differentiating metastatic PDAC from ICA by comparing their expression between these two groups of cancer tissues.

## RESULTS

### The clinical and pathological features

The clinicopathological information gathered from PDAC, chronic pancreatitis and cholangiocarcinoma patients and their matched healthy controls in the ELISA and IHC studies are summarized in Tables 1 and 2. There were no statistical differences among the groups in terms of gender and age distribution, lymphatic invasions, and metastasis. However, PDAC cases showed significantly more advanced tumor stages than ICA and ECA cases, corresponding directly with their more aggressive behavior.

### Reg proteins were involved in PDAC precursors including ADM and PanIN lesions

#### Reg1A and Reg3A/G were involved in acinar-to-ductal metaplasia

**Figure 1:**
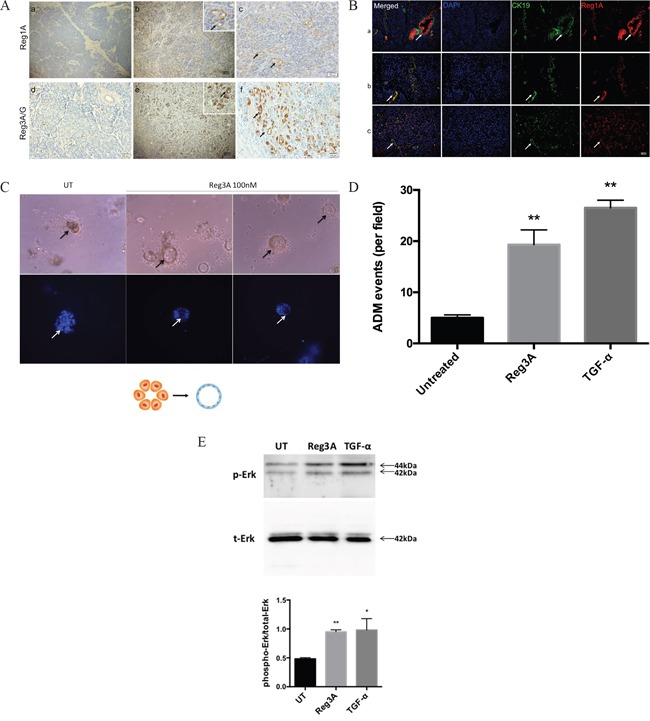
Reg1A and Reg3A/G (pancreatitis-associated proteins) were associated with ADM (A) Increased Reg1A and Reg3A/G protein expressions in acinar cells undergoing ADM, compared to normal tissues a, d) normal tissues, b, e) areas undergoing ADM, c, f) magnified pictures showing ADM clusters, positive for Reg1A and Reg3A/G. Arrows: duct-like structure in tumor adjacent acini **B.** Co-localization of Reg1A and CK19 in cancer epithelium and duct-like structures. a) arrows: cancer cells, as positive controls, b, c) arrows: duct-like structures. Blue: DAPI, Green: CK19, Red: Reg1A. **C.** Reg3A (100 nM) promoted the formation of duct-like structure in primary acinar cells in 3-D culture. Arrows: duct-like structures. Bottom: blue DAPI showing cell nuclei. The graphic illustrates the model of ADM formation. **D.** Quantification of the cysts formation from primary acinar cells. Data was presented as cysts formation per imaging field. The images were taken under 100x magnification, and the analysis was performed using Image J software. **E.** Western blotting of the phosphorylated and total Erk1/2 in primary acinar cells after 30 min treatment of Reg3A or TGF-α. The upper panel showed the representative membrane selected from 3 repeats of the experiment. The lower panel was the quantification of phosphorylated Erk corrected by total Erk levels (Image Lab). N=3, *p<0.05, **p<0.01 vs. vehicle treated cells.

ADM is defined as a transdifferentiation of acinar cell to ductal cell phenotypes. It is characterized by the formation of duct-like structures, decreased expression of acinar biomarkers such as amylase, and increased expression of ductal biomarkers such as cytokeratin 19 (CK19). Mounting evidence supports the involvement of ADM in the initiation of PDACs [17]. We screened the expression of all Reg protein isoforms in human PDAC tissues vs. normal tissues. No expression of Reg1A or Reg3A/G was observed in normal acini and ducts (Figure 1A). However, Reg1A and Reg3A/G positive duct-like structures were observed in tumor-adjacent acinar areas (Figure 1A). To validate the ductal phenotypes of these structures, CK19 was co-stained with Reg1A using immunofluorescence, confirming that the Reg protein positive structures were ADM (Figure 1B).

To directly study the role of Reg proteins in promoting ADM, we established a 3-D culture model for primary acinar cells *in vitro* by using matrigel. Acinar cells treated with Reg3A exhibited increased duct-like cysts formation (19.3±2.9% cysts per visual field), comparable with the positive control, TGF-α treated cells (26.5±1.5%). However, cells treated with vehicles still remained as acinar clusters, with only 5% sporadic cysts formation. (Figure 1C, 1D). This suggests that Reg3A could promote the transition of acinar cell to ductal cell phenotypes.

To understand the underlying mechanism, primary acinar cells were treated with Reg3A or TGF-α for 30 min and the status of Erk phosphorylation was assessed using Western blotting. Compared with vehicle-treated cells, Reg3A-treated cells showed a significantly higher level of Erk phosphorylation (Figure 1E), comparable with the positive control TGF-α treated cells. This data suggests that the promoting effect of Reg3A on ADM may involve an activation of mitogen-activated protein kinase (MAPK), which is known to mediate the effect of TGF-α.

#### Reg1A and Reg1B were highly expressed in PanIN lesions

**Figure 2:**
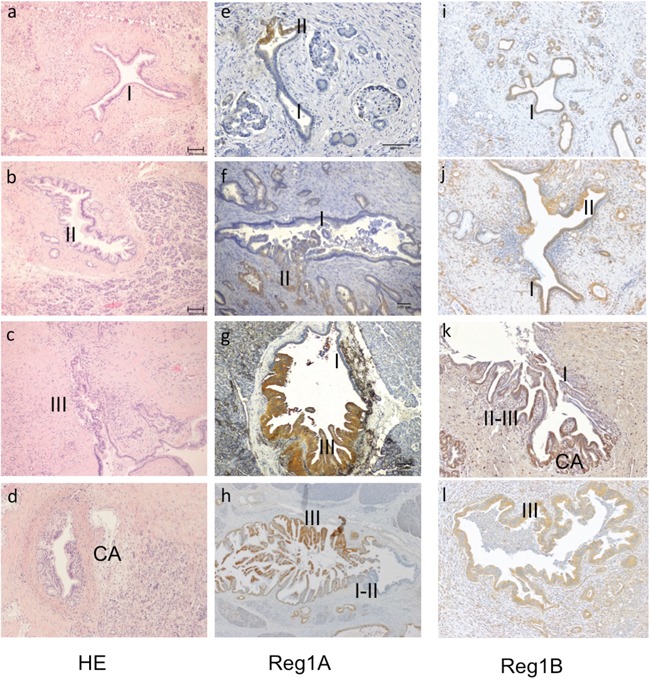
Association of Reg1A and 1B tissue expressions with the histological grades of PanIN lesions and invasive PDACs **A-C.** Representative illustrations of the different grades of PanIN lesions, from PanIN-I, II to III. **D.** Invasive cancer adjacent to a PanIN-II lesion. I-III: PanIN I, II and III; CA: cancer (100x). **E-H.** The staining intensity of Reg1A was positively associated with different grades of PanIN, marked as I, II, III. **I-L.** Reg1B staining was strongly positive with different grades of PanIN seen in ducts. Representative images were selected from at least 10 fields, each at 100x magnification.

Pancreatic intraepithelial neoplasia (PanIN) is the most common precursor to PDAC with four histological grades (IA, IB, II and III), based on the degree of cytological and architectural atypia (Figure 2A-2D) [18]. Immunohistochemically, no expression of Reg proteins was found in normal duct epithelium. As PanIN progresses from low to high histological grades, stepwise increases of Reg1A staining intensity were observed (Figure 2E-2H), whereas Reg1B expression remained elevated during the whole progression from PanIN to PDAC (2I-2L). Different grades of PanIN lesions were observed within the same duct, and positively correlated with the staining intensity of Reg1A (Figure 2G).

### Reg proteins act as diagnostic biomarkers for PDAC

#### Elevation of the serum Reg protein levels in PDAC patients

**Figure 3:**
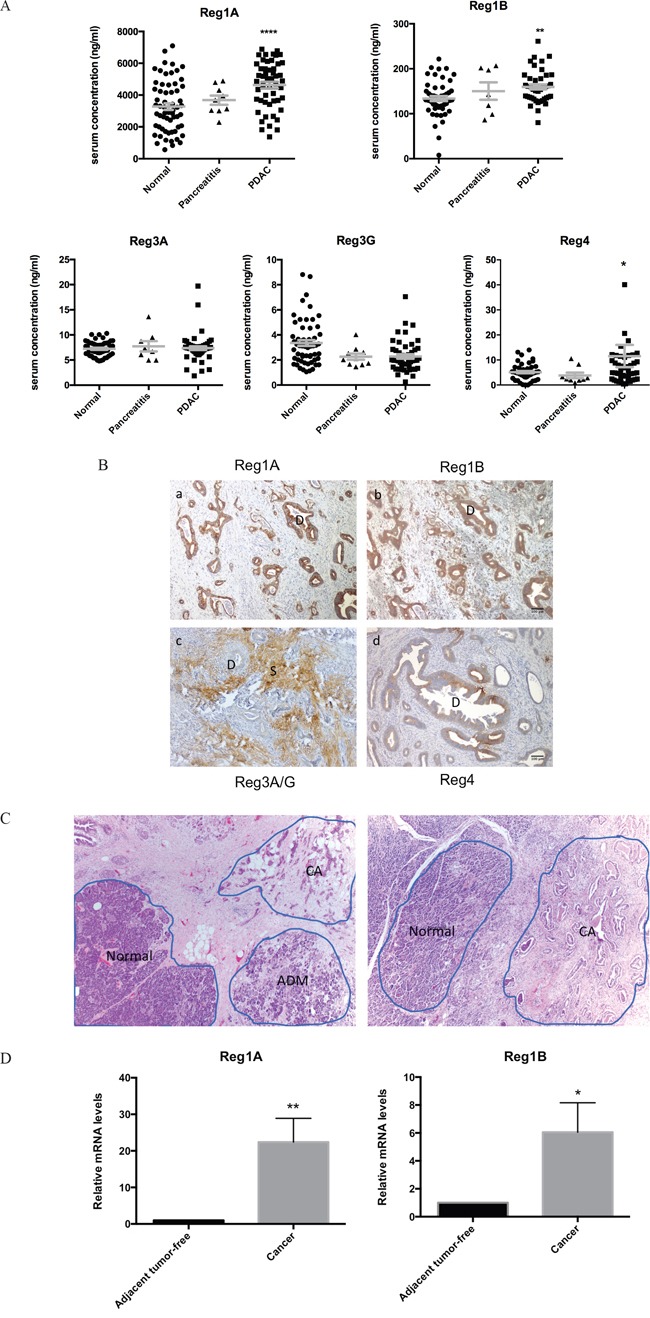
Upregulation of Reg1A and Reg1B in the sera and tissues of PDAC patients **A.** Serum Reg1A and 1B levels in PDAC patients (n=41), healthy controls (n=61) and chronic pancreatitis pateints (n=9). The concentrations of Reg protein isoforms were determined using ELISA. The comparisons were performed using One-Way ANOVA. *P<0.05, **P<0.01, ****p<0.0001. **B.** Immunohistochemistry profiles of Reg proteins in pancreatic ducts and stroma in PDAC patients. The color brown represents positive staining of various Reg protein isoforms. D: ducts, S: stroma. Representative images were selected from at least 10 fields each. **C.** Microdissection of tissues from cancer and adjacent normal areas. Representative images showing how the microdissections were done. ADM areas were excluded for this study. **D.** Relative mRNA levels of Reg1A and 1B in microdissected cancer tissues vs. adjacent tumor-free tissues. N=5; 7. Levels of mRNA in cancer tissues were calculated as fold changes, compared to those in their paired adjacent tissues. *P<0.05, **p<0.01.

Reg proteins are known to be secreted into the circulation under certain conditions [19, 20]. We performed a whole panel of Reg proteins ELISA on the sera of PDAC patients and matching healthy subjects, as well as chronic pancreatitis patients. In comparison to matched healthy subjects, PDAC patients showed significantly higher serum levels of Reg1A, Reg1B and Reg4 (Figure 3A). The differences in serum Reg4 levels appeared less dramatic than those of Reg1A and Reg1B. There were only slight elevations of Reg1A and Reg1B levles in the sera of chronic pancreatitis compared to the normal controls which were not statistically significnant (Figure 3A, mid column). Finally, there were no statistically significant differences in the serum levels of Reg3A or 3G between PDAC, chronic pancreatitis patients, and matching healthy individuals.

#### Increased expression of Reg proteins in PDAC tissues

Immunohistochemically, the infiltrative PDAC cancer glands stained strongly positive for Reg1A and Reg1B, but negative for Reg3A/G (Figure 3B). The intensity of staining for Reg1A and Reg1B in these cancer cells was much stronger than that of Reg4 (Figure 3B), despite the fact that Reg4 had previously been reported as a biomarker of PDAC [14, 21–23]. Unlike the strong and evenly distributed staining of Reg1A and Reg1B in malignant glands, the staining of Reg4 was uneven and varied from mild to intermediate and strong in some areas of the malignant glands. Distinct from other isoforms, Reg3A/G showed strong staining exclusively in the stroma, which is composed of extracellular matrix proteins, stellate cells, fibroblasts, and lymphocytes. Increased stromal Reg3A/G expression may contribute to the reduced penetration of chemotherapy drugs in the cancer tissue, and associated with drug resistance [24].

#### Increased Reg1A and Reg1B mRNA levels in the PDAC tissue

To further validate the serological and immunohistochemical findings, Reg1A and Reg1B mRNA levels were measured in microdissected cancer tissues and adjacent non-neoplastic acinar tissues (Figure 3C). PDAC cancer tissues showed 22-fold and 6-fold increases of Reg1A and Reg1B mRNA levels than the adjacent non-neoplastic acinar tissues (P=0.025 and 0.016; Figure 3D). None of the other isoforms showed significant changes (data not shown).

### Reg1A and 1B act as prognostic biomarkers for PDAC

#### Serum levels of Reg proteins negatively correlated with the histological grades of PDAC

**Figure 4:**
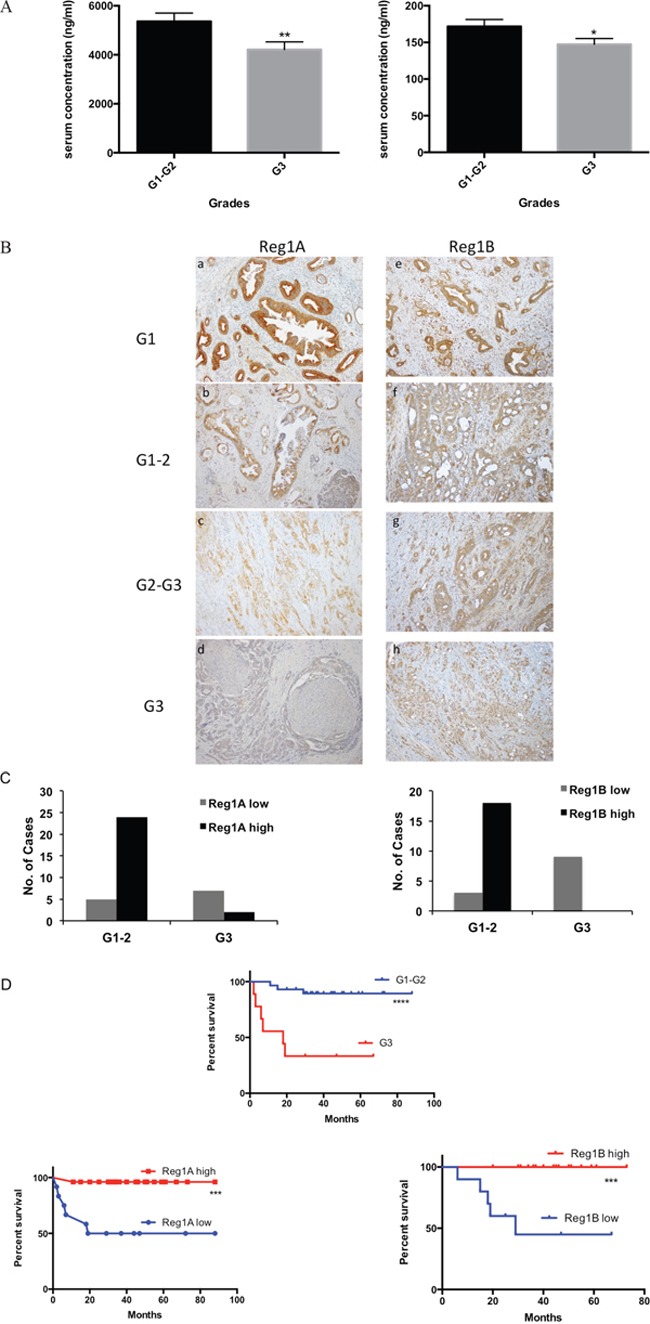
Higher levels of Reg1A and Reg1B were associated with lower differentiation grades of cancer cells and predicted better prognosis **A.** Serum levels of Reg1A and Reg1B were negatively correlated with cell differentiation grades in PDAC patients. Correlation analysis was done by Spearman's test *P<0.05, **P<0.01. **B.** Immunohistochemistry showing Reg1A and Reg1B expression in different differentiation grades. G1: well-differentiated cancer, G2: medium-differentiated cancer, G3: poorly-differentiated cancer. **C.** Positivity of Reg1A and 1B in low and high grades of PDAC. Cases were divided into high and low expression groups, based on their immunohistochemical staining intensity. IRS≥9 was considered as high expression. G1-2 was defined as a low differentiation grade; G3 was defined as a high differentiation grade. **D.** Survival rate of patients with low and high Reg expression levels and differentiation grades. Data were analyzed in GraphPad Prism 6.0. ***P<0.001, ****P<0.0001.

To study whether the serum levels of Reg proteins could predict the malignant progression of PDAC, we performed a correlation analysis between Reg protein serum levels and PDAC clinical stages, grades and metastatic profiles. Interestingly, negative correlation between the histological grades of PDAC and serum Reg1A and Reg1B levels were observed (p=0.003 and 0.028, Figure 4A, Supplementary Table S1). The correlation data generated for other isoforms of Reg protein and other clinical parameters did not exhibit any statistical significance (Supplementary Table S1).

#### Tissue expressions of Reg proteins negatively correlated with the histological grades of PDAC

The negative correlation between the histological grades of PDAC and the serological levels of Reg1A and Reg1B were further validated by the IHC data in tissue samples. There was a gradual decrease pattern of Reg1A and Reg1B expression as PDAC progressed from well and moderate differentiation to poor differentiation (Figure 4B). In low differentiation grades (G1-G2), 83% of the cases showed high Reg1A expression levels and 86% showed high Reg1B expression levels, while in high differentiation grades (G3), 86% and 100% of the cases showed low Reg1A and Reg1B levels (P<0.01 and P<0.0001, Figure 4C), respectively.

#### Higher expression of Reg proteins was correlated with better patients' survival rates

The clinical relevance of the negative correlation between tissue expressions of Reg proteins and histological grades of PDAC was analyzed by incorporating patients' survival data. High grade PDAC with low levels of Reg1A and Reg1B showed a statistically significant lower survival rate after tumor resection, when compared to those with low grade PDAC and high levels of Reg1A and Reg1B (P<0.0001, P<0.001 and P<0.001, Figure 4D). It suggests that the prediction value of Reg proteins in PDAC patient survival rate is dependent on their histological grades.

### Reg proteins can clearly differentiate ICA from PDAC

**Figure 5:**
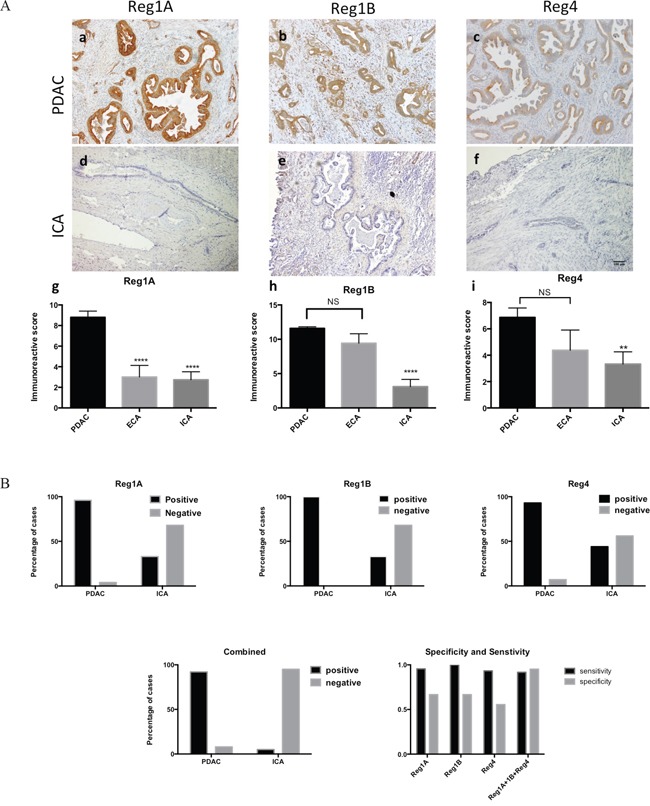
Tissue expression of Reg1A, Reg1B, and Reg4 could differentiate PDAC from ICA with high specificity and sensitivity **A.** Immunoreactive scores (IRS) of Reg1A, Reg1B and Reg4 in PDAC were significantly higher than those in ICA. a-c) Reg1A, Reg1B and Reg4 expression in PDAC; d-f) Reg1A, Reg1B, and Reg4 expression in ICA; g-i) comparisons of Reg1A, Reg1B, and Reg4 IRS among PDAC, ECA, and ICA. **P<0.01, ****P<0.0001, NS: no significance. **B.** The sensitivity and specificity of combining Reg1A, Reg1B, and Reg4 immunohistochemical staining in distinguishing PDAC from ICA. Positive and negative cases of PDAC and ICA for Reg1A, Reg1B, and Reg4 were plotted on column graphs. Combined positivity was recorded when Reg1A, Reg1B, and Reg4 were all positive. Sensitivity, specificity and predictive values were calculated by using GraphPad Prism.

Unlike in PDAC, the expression of Reg proteins has not been reported in hepatic cholangiocarcinoma. To investigate whether the tissue expression of Reg proteins could be used to differentiate ICA from metastatic PDAC in the liver, we compared the immunohistochemical expression of Reg proteins in 60 PDAC, 27 ICA and 13 ECA patients. Reg proteins, especially Reg1A and Reg1B, were clearly overexpressed in the PDAC cases, but absent in the ICA cases (Figure 5A, a-c and d-f). The mean scores of Reg1A, Reg1B, and Reg4 in PDAC were 3.2, 3.7, and 2.1 fold of those in ICA, respectively (Figure 5A, g-i). Additionally, the mean scores of Reg1B and Reg4 in ECA did not show any statistical difference with those in PDAC, indicating the closer relations of ECA with PDAC, as compared to ICA.

Based on previous studies, a cut-off value of 2 was determined for categorizing positive from negative IHC results [25]. Among 60 cases of PDAC, 95.6% were Reg1A positive, 100% were Reg1B positive, and 93.3% were Reg4 positive. However, only 33.3% of the ICA cases were Reg1A and Reg1B positive; and 44.4% were Reg4 positive (Figure 5B). Used independently in the differential diagnoses of PDAC and ICA, Reg1A, Reg1B or Reg4 demonstrated high sensitivity (95.56%, 100% and 93.33%, respectively) but low specificity (66.67%, 66.67% and 55.56%, respectively, Figure 5B). However, by combining the scores of all three proteins together, the test specificity increased to 95.24% and the sensitivity remained at 91.67%. The positive and negative predictive values of combining Reg1A, Reg1B and Reg4 in differentiating PDAC from ICA were 0.9524 and 0.8333, respectively.

## DISCUSSION

An increasing number of reports have revealed the contributions of pancreatitis-associated ADM in PDAC initiation [4-6, 26]. As the pancreatitis-associated Reg3 subfamily of proteins are known to contribute to the regeneration of several cell types, it is conceivable that Reg3 may contribute to the transition from pancreatitis to PDAC. Recently, both mouse Reg3β and Reg3g have been reported to promote the transition from chronic pancreatitis to PDAC by using a caerulein-induced pancreatitis mouse model [5, 27]. In this study, we were able to validate at multiple levels, on the involvement of Reg proteins in the ADM process. Firstly, increased Reg1A and Reg3A/G expressions in ADM tissues were demonstrated by immunohistochemistry. Secondly, co-localized expression of CK19 and Reg1A in ADM glands was observed by immunofluorescence. Thirdly, Reg3A treatment can directly induce the formation of ADM in a 3-D culture model *in vitro*. We were able to demonstrate the upregulation of phosphorylated Erk upon Reg3A stimulation, indicating the activation of MAPK pathway. MAPK has been shown to be required for the formation of ADM and PanIN lesions. Its inhibition promotes a re-differentiation of the duct-like cells back to normal acinar cells and prevents the development of PanIN lesions in Kras mutated mice [28]. Therefore, we believe that the induction of ADM by Reg3A is at least partially through MAPK pathway. Interestingly, we have also found that as PanIN advanced from PanIN-1 to PanIN-3, and finally to invasive PDAC, there were stepwise increases in the tissue expressions of Reg1A and Reg1B proteins. Since both ADM and PanIN are established precursors for PDAC, our data supports the notion that there is a close correlation between Reg protein levels and the disease progression from ADM, PanIN to PDAC.

One of the important reasons for the high mortality rate of PDAC is that most patients are at the advanced stages and with very limited therapeutic options at the time of the initial diagnosis. This phenomenon was also reflected in the clinical characteristics of patients in our study group (Table 1 and 2). Sensitive and specific biomarkers could aid in early diagnosis, facilitate effective therapy and improve a patient's prognosis [29]. In this study, we found significant elevations of Reg1A and Reg1B in the sera of pancreatic cancer patients in comparison to normal healthy subjects. We further validated the increases of these two proteins in both mRNA and proteins levels in cancer cells. Our data suggests that serum levels of Reg1A and Reg1B are superior than previously reported Reg 4, and could be used as clinically applicable biomarkers for the early diagnosis of PDAC [14, 15, 30, 31]. We also observed statistically insignificant elevations of Reg1A and Reg1B in the sera of chronic pancreatitis compared to normal controls. These slight elevations could be associated with ADM, which is frequently observed in tissues of pancreatitis patients. These data further support the clinical values of Reg1A and Reg1B as early diagnostic biomarkers. If validated in large-scale, multi-center studies, Reg1A and Reg1B serological testing should be made available as a routine screening test for detecting PDAC in the at-risk patient population, and for monitoring PDAC and metastasis after therapy.

**Table 1:** Clinical information of healthy subjects (N=61), chornic pancreatitis (n=9) and PDAC patients (N=41) whose sera were used for the study of Reg proteins by ELISA

	Healthy (n=61)	Chronic Pancreatitis (n=9)	PDAC (n=41)	P value
Age (years, median, mean± S.E.M.)	56 ± 1.4	63 ± 6.0	59 ± 2.0	0.99
Sex (M: F)	41:20	6:3	27: 14	
Differentiation Grades				
G1	-	-	0	
G2	-	-	19	
G3	-	-	22	
Lymphatic invasion				
Absent	-	-	26	
Present	-	-	15	
Metastasis				
Absent	-	-	20	
Present	-	-	21	
TNM staging				
IA/IB	-	-	4	
IIA	-	-	10	
IIB	-	-	5	
III	-	-	3	
IV	-	-	19	

**Table 2:** Clinical information of PDAC, ECA and ICA patients whose tissues were used for the immunohistochemistry study

	PDAC (n=60)	ECA (n=13)	ICA (n=27)	P value
Age (years, mean± S.E.M.)	69±1.3	60±1.8	62±2.3	#0.0004 ***
Sex				
M	48	10	18	0.37
F	12	3	8	
Differentiation Grades (%)				
G1	3	0	3	0.47
G2	45	10	21	
G3	12	3	3	
Lymphatic invasion (%)				
Absent	14	6	12	0.12
Present	46	7	15	
Metastasis (%)				
Absent	56	12	27	0.40
Present	4	1	0	
TNM staging				
IA/B	5	5	6	0.0017**
IIA	9	2	8	
IIB	41	4	6	
III	4	1	3	
IV	1	0	4	

# Data analysis was done by using a one-way ANOVA. All the other analyses were done by using a Chi-square test.

*P<0.05

** P<0.01

*** P<0.001.

To our surprise, both the serum levels and tumor tissue expression of Reg1A and Reg1B showed negative correlations with the histological grades of invasive PDAC. This observation suggests that a certain level of pancreatic duct differentiation may be required for the sufficient expression and secretion of Reg1A and Reg1B proteins. Loss of ductal differentiation will likely cause a decrease in Reg protein levels. Clinically, low grade tumors tend to have larger tumor volumes when they are diagnosed, and consequently may have the capability to secrete more Reg proteins into the circulation. In our study population, patients with poorly differentiated PDAC and low levels of Reg1A and Reg1B demonstrated higher mortality than those with well to moderately differentiated PDAC and high levels of Reg1A and Reg1B. The association between poor survival and high histological grade PDAC was established in previous studies [32, 33]. Therefore, the negative correlation between serum Reg1A and Reg1B levels and patients' survival is believed to be dependent on the histological grades. Our observation suggests that Reg proteins could be used as prognostic biomarkers for PDAC patients. Furthermore, this observation also indicates that well- to moderately-differentiated tumors may be more sensitive to Reg protein-targeted therapy than poorly differentiated tumors.

As PDAC progresses, it can metastasize to the regional lymph nodes, liver and other less common sites of the body. When PDAC metastasizes to the liver, it needs to be differentiated from ICA. This clinical demand typically presents itself in the following two scenarios: through the execution of a small liver mass biopsy, and when a small lesion is found in the liver during surgery for a pancreatic tumor. Both scenarios are nightmarish for pathologists because the histological and immunohistochemical profiles of ICA and PDAC are essentially identical. Recognizing the clinical importance of differentiating ICA from PDAC and their associated pathological challenges, dozens of studies have attempted to resolve this issue from different angles. Collins et al. attempted to use microRNA profiling to differentiate these two cancers and found that 15 microRNAs were dysregulated in both tumor types, with seven of them demonstrating opposite expression patterns between ICA and PDAC [34]. Hooper et al. showed that HPC2 expression was observed in 80% of PDACs, and 32% of ICAs, while N-cadherin antibody stained 27% of the PDAC resections versus 58% of the ICA resections [35]. Recently, a novel technology using branched DNA-enhanced albumin RNA in situ hybridization was reported to distinguish hepatocellular carcinoma and ICA from metastatic PDAC [36, 37]. Although promising, none of these previous studies have found their way into the current routine clinical practice due to their low specificity or sensitivity, or technical complexity. By contrast, our study demonstrated the highest sensitivity and specificity (92% and 95%, respectively) to date, and the combination of Reg1A, Reg1B, and Reg4 IHC staining is easy to conduct compared with previous studies [34, 35, 38, 39]. The only limitation of this study is its relatively limited case numbers. However, the differences observed in this study strongly suggest the potential applicability of Reg proteins in routine clinical practice and warrant further validation in larger, multi-institutional studies.

**Figure 6:**
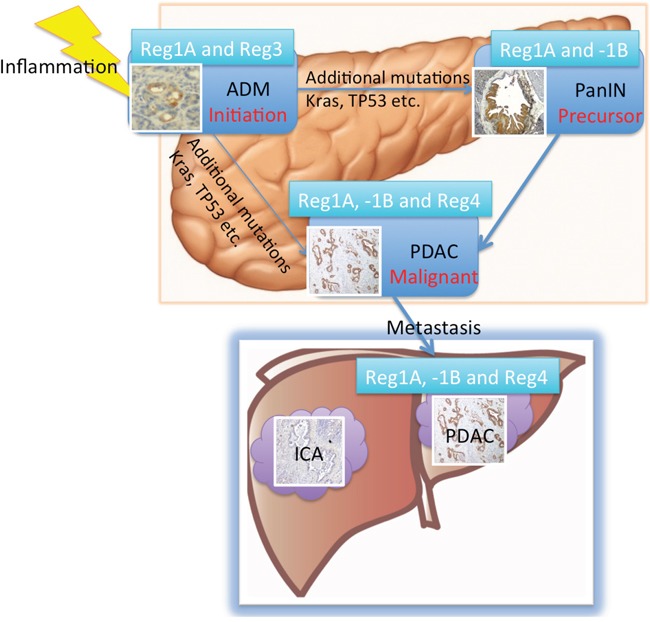
Summary of the roles of Reg proteins in the progression of ADM/PanIN to invasive cancer and metastasis in liver The expression levels of Reg1A and Reg3A/G were elevated in ADM. Reg3A seems to directly promote ADM formation *in vitro*. The effects of additional mutations such as in Kras and TP53 transform ADM further into PanIN lesions and PDAC [43]. Serum levels of Reg1A and 1B as well as their tissue expressions were all elevated in PDAC patients. As such, increased Reg proteins may be used to differentiate PDAC from ICA.

In summary (Figure 6), this is the first systematic assessement of the diagnostic and prognostic values of all Reg protein isoforms using serological, immunohistochemical and molecular technologies in close correlation with clinical follow-up data. We have shown that the expression of Reg proteins in the precursor lesions of PDAC (ADM and PanINs). In the 3-dimensional culture of acinar cells, Reg3A promoted ADM formation through the activation of MAPK pathway. The combination of Reg1A, Reg1B, and Reg4 tissue expressions could clearly differentiate metastatic PDAC from ICA with very high sensitivity and specificity.

## MATERIALS AND METHODS

### Patients and tissue samples

Tissues samples of PDAC (n=60), intrahepatic cholangiocarcinoma (ICA) (n=27), and extrahepatic cholangiocarcinoma (ECA) (n=13) were obtained from patients who underwent resections at the Department of Pathology at the University of Calgary (Canada), the Beijing You An Hospital (China), and McGill University (Canada) between 2009 to 2014. The tumor diagnoses, histological grades and stages were reassessed by two pathologists (ZHG and KD). Ampullary adenocarcinoma, duodenum adenocarcinoma, mucinous adenocarcinoma, hepatocellular carcinoma, and bile duct adenoma were excluded from the study. Clinical information was obtained upon the original pathology requisitions and physician's notes in the charts. The study was approved by the institutional Research Ethics Board (REB) [40].

### Serum samples

Peripheral blood samples were obtained from 61 healthy donors, 9 chronic pancreatitis and 41 PDAC patients at the Affiliated Hospital of Qingdao University and McGill University Health Center between September 2012 and March 2015. Samples were centrifuged for 10 min at 1000×g under 4°C, and the serum was stored in cryovials at −80°C until examination. All the PDAC patients involved in this study were newly diagnosed before any medical treatment or surgery.

### Immunohistochemistry and immunofluorescence

For the detection of Reg proteins in human PDAC tissue samples, 4 μm tissue sections were deparaffined in xylene and rehydrated in graded ethanol. 0.1% Triton X-100, and 3% hydrogen peroxide were used prior to 10% blocking serum. Sections were incubated overnight with primary antibodies (hReg1A, hReg1B and hReg3A/G from Santa Cruz, TX, USA; hReg4 from R&D, MN, USA). Corresponding secondary antibodies were administrated, followed by the addition of DAB (SK-4105, Vector, CA, USA). The slides were then counterstained with hematoxylin (Thermo Fisher, MA, USA) and mounted with hydrophobic medium. For immunofluorescence, Cytokeratin 19 (TROMA-III, DSHB) and Reg1A antibodies were used to detect the colocalization of these two markers on the tissues that had undergone acinar-ductal metaplasia in PDAC patients. Corresponding Alexa Fluor dyes were used for fluorescent detection. DAPI was used for nuclear counter staining.

### Evaluation of Reg proteins immunostaining

The immunohistochemical staining on whole slides was independently evaluated under 100x magnifications by two pathologists. The immunoreactive scoring (IRS) system that evaluated the proportion of positivity and the intensity of staining was adapted from previous work on Reg4 and other proteins [41, 42]. The percentage of positive tumor cell staining was scored as 0 (negative), 1 (<25%), 2 (26-50%), 3 (51-75%), and 4 (>75%). The intensity of tumor cell staining was graded 0 (negative), 1 (light yellow color), 2 (brownish-yellow), and 3 (brown). Grades 0-1 were defined as low expression, and 2-3 were defined as high expression. The two scores were multiplied, and the final IRS value was determined. A final score equivalent to <2 was considered negative. Compound positivity was recorded when Reg1A, Reg1B and Reg4 were all positive.

### Three-dimensional culture and Western blotting

Primary acinar cells were isolated from C57BL/6 mice by Collagenase P (Sigma). Cells were then suspended in 5% matrigel/medium (v/v) and seeded on 8-chamber slides pre-coated with matrigel (Cat No. 354248, Corning). Reg3A (100 nM, mouse isoform, Biomart) was added to the RPMI 1640 culture medium containing 10% fetal bovine serum (FBS), soybean trypsin inhibitor (0.1mg/ml, Sigma) and dexamethasone (1 μg/ml, Sigma). The medium was changed every 2 days and cells were cultured up to 14 days. DAPI was used to stain the nuclei. Pictures were taken under inverted microscope.

To determine the activity of Erk, primary acinar cells were cultured in the medium containing 2% FBS. Cells were collected after 30 min of treatments. Western blot was performed to determine the levels of phosphorylated and total Erk1/2 (sc-16982R and sc-154, Santa Cruz, TX, USA). Pictures were taken by using ChemiDoc Touch Imaging System (Bio-Rad).

### Enzyme-linked immunosorbent assay (ELISA)

Reg protein (Reg1A, 1B, 3A, 3G and 4) concentrations were determined by ELISA (Uscn Life Science Inc. China). An Avidin-Biotin system was used to develop colors and changes were measured at a wavelength of 450 nm (Perkin-Elmer, Enspire 2300, MA, USA). Results were expressed as ng/ml.

### Microdissection and quantitative RT-PCR in PDAC tissue vs. paired adjacent non-neoplastic tissues

Representative tissue blocks were selected to perform microdissection based on H&E staining in FFPE tissue sections (15μm-thick, 10 consecutive slides). Total RNA was prepared by using RecoverAll Total Nucleic Acid Isolation Kit for FFPE (AM1975, Ambion). Quantitative real-time PCR was performed by using PowerUp SYBR Green Master Mix (A25742, Applied Biosystems). Primers for Reg1A, Reg1B and GAPDH were designed and synthesized from Life Technologies (sequences listed in Supplementary Table S2). The relative expression levels were normalized by GAPDH, and fold changes were calculated by comparing cancer vs. its paired tumor-adjacent non-neoplastic tissues.

### Statistical analysis

All data were expressed as Mean ± SEM. One-Way ANOVA and student's two-tailed t-test were used for comparison of ELISA, qPCR and immunoreactive scores results. The correlation analyses were done by using Spearman's test. Chi-square test and Fisher's exact test were used for comparisons of Reg protein expression in PDAC and ICA. The sensitivity, specificity, and predictive values of combining Reg1A, Reg1B, and Reg4 immunohistochemical staining for differentiating ICA from PDAC were calculated using GraphPad Prism 6.0 program. P<0.05 was considered as statistical significant. Data management was performed by using the GraphPad Prism 6.0 and SPSS statistics software (version 21).

## SUPPLEMENTARY MATERIALS TABLES


