# Supplementary material for: Reg proteins promote acinar-to-ductal metaplasia and act as novel diagnostic and prognostic markers in pancreatic ductal adenocarcinoma
Source: Oncotarget. 2016 Oct 24;7(47):77838–53. doi: 10.18632/oncotarget.12834 (PMC5363625; doi:10.18632/oncotarget.12834)
Supplement: Supplementary file 1 [file oncotarget-07-77838-s001.pdf]

Reg proteins promote acinar-to-ductal metaplasia and act as novel diagnostic and prognostic markers in pancreatic ductal adenocarcinoma

SUPPLEMENTARY TABLES

Supplementary Table S1: Correlation of serum Reg proteins levels with TNM staging and histology grading of PDAC. N=41

		Reg1A	Reg1B	Reg3A	Reg3G	Reg4
**TNM stages**	Correlation Coefficient	0.101	0.313	-0.158	-0.191	0.141
	P value	0.530	0.047*	0.324	0.232	0.378
**Differentiation Grades**	Correlation Coefficient	-0.446	-0.343	-0.273	0.004	0.095
	P value	0.003**	0.028*	0.084	0.980	0.554

The statistical analysis was performed by using a Spearman's test. *P<0.05.

Supplementary Table S2: Nucleotide sequences of primers used for quantitative PCR

Gene name	Forward Primer	Reverse Primer
Reg1A	GAGAAGCCAACTCAGACTCAG	TGAGACAGAAACATCAGGCAG
Reg1B	GGTCCTGCAATTACTATGAAGTCAAA	AAGATCAGCGATGCAAACTCATT
GAPDH	TGACAACTTTGGTATYCGTGGAAGG	AGGCAGGGATGATGTTCTGGAGAG
